# Health professional students’ perceptions and preparedness for interprofessional education: a multicentric analysis across five countries

**DOI:** 10.3389/fmed.2025.1665243

**Published:** 2025-09-01

**Authors:** Thani Alsharari, Osama Khattak, Anshoo Agarwal, Farooq Ahmad Chaudhary, Nida Suhail, Gulam Saidunnisa Begum, Geetha Subramaniam, Hathal Albagami, Mohammed Fareed Felemban, Hassan Talat Shawli, Fahad Saeed Algahtani, Muhammad Amber Fareed

**Affiliations:** ^1^Department of Restorative Dental Science, Faculty of Dentistry, Taif University, Taif, Saudi Arabia; ^2^Department of Restorative Dentistry, Jouf University, Sakaka, Saudi Arabia; ^3^Pathology Department, Faculty of Medicine, Northern Border University, Arar, Saudi Arabia; ^4^School of Dentistry, Shaheed Zulfiqar Ali Bhutto Medical University, Islamabad, Pakistan; ^5^Department of Medical Laboratory Technology, Faculty of Applied Medical Sciences, Northern Border University, Arar, Saudi Arabia; ^6^Department of Biochemistry, College of Medicine and Health Sciences, Suhar Campus, National University, Muscat, Oman; ^7^Faculty of Health and Life Sciences, INTI International University, Nilai, Malaysia; ^8^Department of Restorative Dental Sciences, Faculty of Dentistry, Taibah University, Madinah, Saudi Arabia; ^9^Department of Oral and Maxillofacial Surgery and Diagnostic Sciences, Faculty of Dentistry, Taif University, Taif, Saudi Arabia; ^10^Clinical Sciences Department, College of Dentistry, Ajman University, Ajman, United Arab Emirates; ^11^Center of Medical and Bio-allied health Sciences Research, Ajman University, Ajman, United Arab Emirates

**Keywords:** interprofessional collaboration, interprofessional education, effective communication skills, RIPLS, health professional

## Abstract

**Introduction:**

Interprofessional education (IPE) prepares health professional students for teamwork in clinical practice. The success of IPE depends on students’ readiness and acceptance of interprofessional learning. This study aimed to assess the perceptions and readiness for IPE among health professional students across universities in five countries, using the Readiness for Interprofessional Learning Scale (RIPLS).

**Methods:**

A cross-sectional descriptive study was conducted among 335 students from health professional programs at universities in Saudi Arabia, Malaysia, Nepal, India, and Pakistan. The Readiness for Interprofessional Learning Scale (RIPLS) questionnaire was used to collect data prior to any structured workshop activities, and results represent baseline perceptions of IPE. Responses were analyzed using descriptive statistics, paired-samples t-tests, and analysis of variance (ANOVA) to compare groups.

**Results:**

The study found significant differences (*p* < 0.001) in IPE readiness between students from different countries and academic years. Students from Malaysia, India, and Pakistan demonstrated higher readiness for IPE, with senior students showing greater readiness compared to juniors. The mean readiness score for teamwork and collaboration was 4.2 (SD = 0.7) in Malaysia, while students in Nepal had the lowest mean score of 3.2 (SD = 1.5). Overall, senior students exhibited higher readiness for teamwork and collaboration (mean = 4.3, SD = 0.4) compared to first-year students (mean = 3.6, SD = 1.9).

**Conclusion:**

The findings underscore the need for targeted IPE initiatives that are adapted to different academic levels and contexts. Integrating IPE early in healthcare curricula is critical for enhancing collaborative skills and improving patient outcomes. These results have important implications for curriculum developers and policymakers aiming to foster interprofessional collaboration in healthcare education.

## 1 Introduction

Interprofessional collaboration (IPC) has emerged as a vital strategy for improving healthcare delivery and patient outcomes (1, 2). In response, health professional education programs are increasingly designed to equip graduates with the necessary skills to collaborate effectively across professions (3). Interprofessional education (IPE), defined as “an experience that occurs when students from two or more professions learn about, from, and with each other” (4, 5), has proven effective in preparing students for IPC by fostering teamwork, enhancing collaborative behavior, and minimizing clinical errors (6–8). For IPE to achieve its goals, students must demonstrate both readiness and willingness to engage in interprofessional learning alongside peers from diverse professional backgrounds (9, 10). In the context of interprofessional education, “readiness” refers to a student’s willingness, openness, and preparedness to engage in learning with peers from other health professions. It encompasses attitudes toward collaboration, role understanding, and appreciation of team-based care. ‘Perception’, on the other hand, relates to students’ beliefs, views, and judgments about the value, relevance, and potential benefits of interprofessional learning in their academic and future clinical practice. However, the extent to which students’ attitudes and readiness align with the goals of IPE remains underexplored in many regions. A deep understanding of students’ pre-existing attitudes and perspectives is essential for developing and implementing effective educational interventions, as well as assessing the efficacy of IPE initiatives (11). In numerous countries, accreditation bodies now mandate IPE experiences for health science students (12, 13). However, integrating IPE into existing professional curricula presents substantial challenges. It requires careful planning and evaluation to ensure students are well-prepared for collaborative learning and to support educators in delivering high-quality IPE (14).

Key aspects of health professional students’ attitudes toward IPE include their readiness for clinical practice and perceptions of preparedness. These dimensions can be assessed through curriculum evaluations, skill proficiency, and simulation training, which help gauge students’ preparedness for real-world clinical scenarios. Surveys, focus groups, and mentorship programs offer valuable insights into students’ experiences and concerns regarding clinical readiness. Mentorship, in particular, plays a vital role in fostering students’ confidence in their ability to collaborate effectively within interprofessional healthcare teams.

Despite global momentum, there is a lack of multicentric evidence from developing regions on how students perceive and engage with interprofessional education (IPE). This study addresses that gap by assessing students’ readiness across five countries where IPE is under-researched. By exploring cross-national differences and contextual influences, the study contributes to building a more globally inclusive understanding of IPE implementation (15). This gap underscores the importance of assessing the relevance and applicability of IPE instruments and assumptions based on experiences from developed regions, especially when addressing the unique challenges faced by developing nations (15). To design and execute IPE activities that yield positive outcomes, it is crucial to evaluate students’ readiness to engage in interprofessional learning, focusing on key IPE dimensions, including the benefits to patient care, health systems, and individual practitioners, as well as teamwork and collaboration among professional groups (16). The rationale for this study is to address the gap in the literature concerning how students from developing countries perceive and engage in IPE. By focusing on five countries, India, Pakistan, Nepal, Malaysia, and Saudi Arabia, this study hopes to provide a comprehensive understanding of health professional students’ readiness for IPE in regions where such data are scarce. Therefore, this study aimed to examine the readiness and perceptions of health profession students from five countries, India, Pakistan, Nepal, Malaysia, and Saudi Arabia, regarding IPE before the implementation of IPE courses and activities.

## 2 Methods

This cross-sectional, descriptive study utilized a convenience sampling approach to enroll undergraduate students from healthcare-related colleges to evaluate their perspectives on the benefits and challenges of interprofessional education (IPE). Students who had graduated from or withdrawn from the program for more than a year were excluded from participation. All students were informed about the study’s objectives, and written consent was obtained in line with ethical guidelines, ensuring confidentiality and anonymity. The sample size was calculated using the Raosoft sample size calculator, which indicated a minimum of 335 students was required to achieve a 95% confidence interval, assuming a total population of 2,300 health college students with a 5% margin of error. To mitigate recall bias, a 10% oversample was added to the final figure. A total of 335 students from health professional colleges, including Dentistry, Medicine, Applied Medical Sciences, Nutrition, and Physiotherapy, across universities in Saudi Arabia, Malaysia, Nepal, India, and Pakistan participated. Local coordinators at each site were responsible for disseminating study information to eligible students via email, institutional platforms, and in-person briefings. Students from various academic years (1st to 6th year) who were currently enrolled in health-related degree programs and had not participated in any formal IPE activity previously were eligible to participate. Participation was voluntary, and all students provided written informed consent before completing the questionnaire. No incentives were offered, and anonymity and confidentiality were maintained throughout the study.

These five countries were selected due to their diverse educational and healthcare contexts, varied stages of IPE integration, and established institutional collaborations that enabled multicentric participation.

The study received ethical approval from multiple review boards: Shaheed Zulfiqar Ali Bhutto Medical University, Pakistan (SOD/ERB/2023/143), Kathmandu University School of Medical Sciences (IRC-KUSMS: 23/031), and University Sains Malaysia (USM/JEPeM/16100380). The study followed the principles of the Declaration of Helsinki and its amendments. Written informed consent was obtained from all participating students after the purpose and objectives of the study were explained. Data were collected through a valid Readiness for Interprofessional Learning Scale (RIPLS) questionnaire developed by Visser et al. (17). This 19-item self-report instrument comprises four subscales: teamwork and collaboration (TC), negative professional identity (NPI), positive professional identity (PPI), and roles and responsibilities (RRs). A 5-point Likert scale was used for each item, ranging from “strongly disagree” (1) to “strongly agree” (5). For the NPI subscale, the scoring was reversed for negative statements (items 10, 11, and 12). The overall possible score for RIPLS ranged from a minimum of 19 to a maximum of 95. The RIPLS tool was used in English. As all participating institutions use English as the primary medium of instruction, no translation was required. However, face validity was piloted on 10 students in each country to ensure clarity and cultural appropriateness. Negative items (10–12) were reverse-coded before analysis. Cronbach’s alpha was computed for the overall scale and found to be 0.90, indicating excellent internal consistency. Subgroup reliability analysis revealed Cronbach’s alpha values ranging from 0.82 to 0.91 across the five country samples. Before administering RIPLS, the internal consistency of each subscale was validated and assessed.

In conjunction with the RIPLS survey, structured virtual workshops were conducted to enhance students’ understanding of the IPE framework and the RIPLS tool. The workshops began with an introduction to IPE and an explanation of the RIPLS tool, including its four subscales. Interactive sessions were then conducted, where students were divided into breakout groups to discuss their experiences with interprofessional collaboration. Role-playing scenarios were used to help students apply the concepts of RIPLS in practical, real-world situations. These workshops served an educational purpose only and were not designed to assess intervention effects. RIPLS responses were collected independently and were not linked to the workshops in a pre- or post-evaluative format.

Data analysis was conducted using the Statistical Package for Social Sciences (SPSS), version 24.0 (IBM, Chicago, Illinois, United States). Descriptive statistics, including means and standard deviations, were used to summarize data. Demographic information was presented as frequencies and percentages. A paired-samples t-test was used to compare pre- and post-intervention results, and analysis of variance (ANOVA) was utilized for comparing more than two groups. The level of significance was set at a *p*-value of < 0.05.

## 3 Results

**Figure 1:**
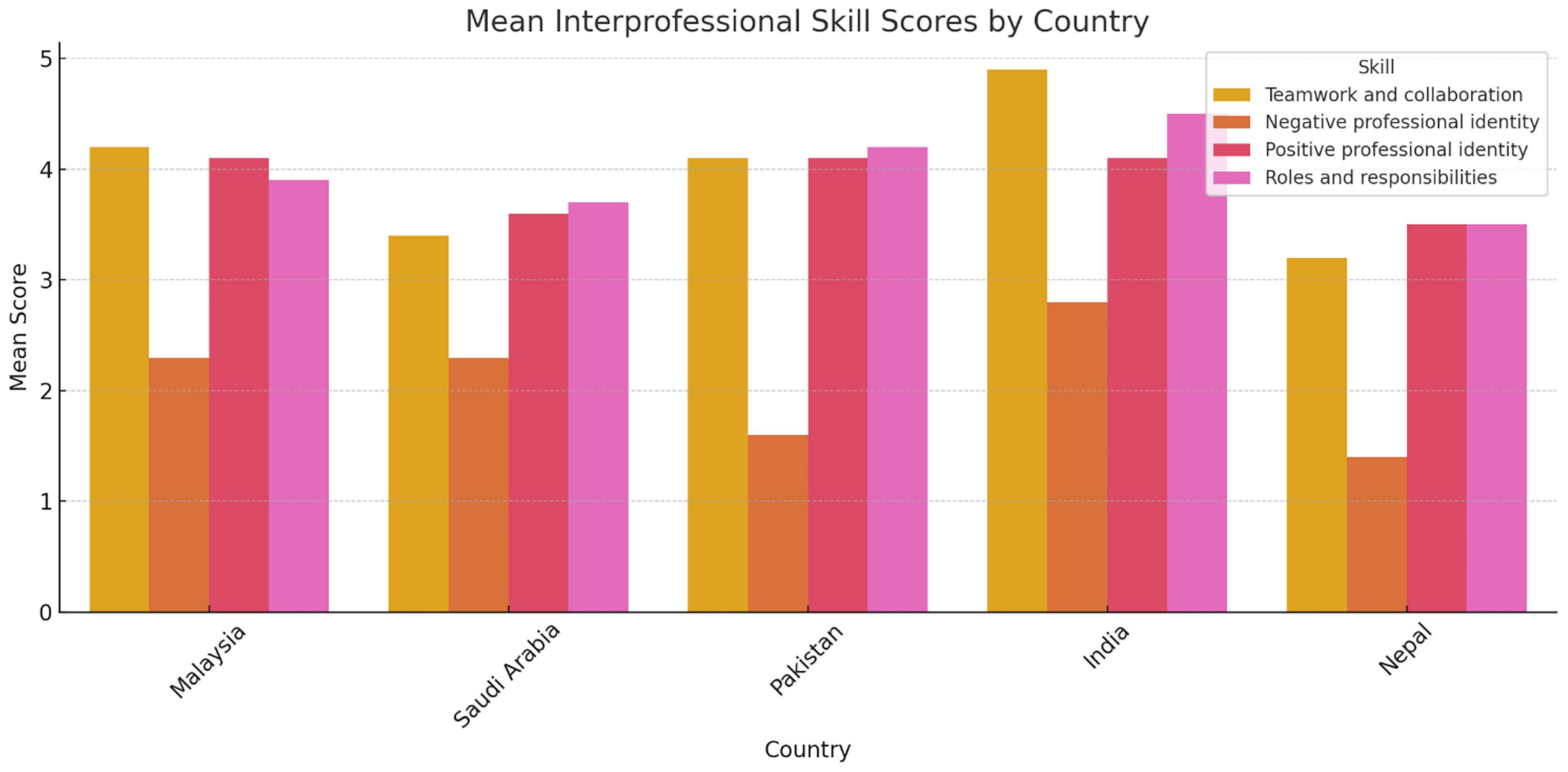
Comparative mean scores for each RIPLS subscale across countries. Malaysia and India showed higher readiness scores in teamwork and collaboration, while Nepal and Saudi Arabia reflected lower scores in certain domains. Group sample sizes were: Malaysia (*N* = 176), Saudi Arabia (*N* = 56), Pakistan (*N* = 56), India (*N* = 17), and Nepal (*N* = 30).

The study used the well-known, validated RIPLS tool to collect data on the readiness and perception of students from different health profession programs across five countries regarding interprofessional learning. Figure 1 illustrates the comparative mean RIPLS subscale scores across all five countries. A reliability study of the collected data revealed high internal consistency (Cronbach’s alpha = 0.90), supporting the findings of this study. A total of 335 students were recruited for the study. The majority of students were female (59.7%), with a mean age of 26 years (SD = 6.0). The largest group of students came from a dentistry program, comprising 40.3% of the sample, followed by medical students (33.4%). Among the five countries represented, the highest percentage of students was from Malaysia (52.5%). The detailed participant demographics are presented in Table 1.

**Table 1:** Demographic details of the students.

Characteristics	No. (%)
Age; mean ± SD	26 ± 6.0
Gender	
Male	135 (40.3)
Female	200 (59.7)
Colleges	
Dentistry	135 (40.3)
Medical	112 (33.4)
Nutrition	16 (4.8)
Applied science	64 (19.1)
Physiotherapy	8 (2.4)
Country	
Malaysia	176 (52.5)
Saudi Arabia	56 (16.7)
Pakistan	56 (16.7)
India	17 (5.1)
Nepal	30 (9)

**Figure 2:**
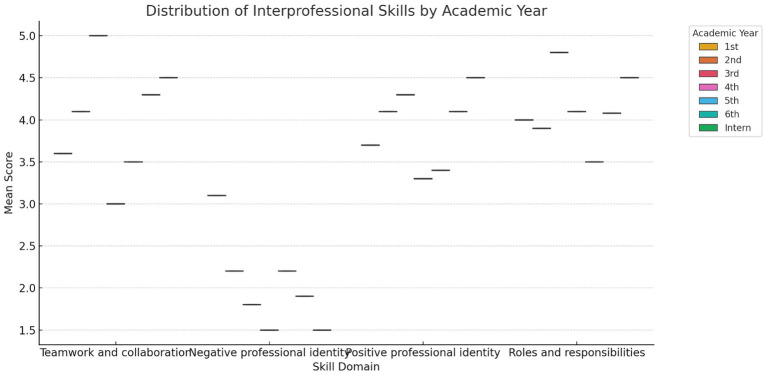
Boxplot shows the distribution of mean RIPLS subscale scores across academic years. Higher academic levels, particularly interns and final-year students, reported stronger readiness in teamwork and roles/responsibilities, while negative professional identity scores declined.

Table 2 presents the mean scores for the individual statements and the overall mean score for each subscale. The statements “Understanding clinical problems better will come from sharing my knowledge with other healthcare students,” “Learning from others will enable me to recognize my limitations,” and “Acquiring knowledge from fellow healthcare students before graduation could enhance connections following graduation” had the highest mean scores, at 4.28, 4.26, and 4.21, respectively. There was a statistically significant difference (*p* < 0.001) between the five colleges across all four interprofessional skills scores: “Teamwork and Collaboration,” “Negative Professional Identity,” “Positive Professional Identity,” and “Roles and Responsibilities” (Table 3). Similarly, there was a statistically significant difference (*p* < 0.001) between all academic years across all four interprofessional skills scores: “Teamwork and Collaboration,” “Negative Professional Identity,” “Positive Professional Identity,” and “Roles and Responsibilities” (Table 4). The analysis revealed significant differences in perceptions of teamwork and collaboration among health professional students from different countries. Students from India (mean = 4.9, SD = 0.2) and Malaysia (mean = 4.2, SD = 0.7) demonstrated the highest levels of readiness for interprofessional teamwork, while students from Nepal reported lower scores (mean = 3.2, SD = 1.5). Similarly, there were variations in positive and negative professional identity, with students from Pakistan (mean = 1.6, SD = 0.48) reporting lower negative professional identity compared to those from Saudi Arabia (mean = 2.3, SD = 1.2). These findings underscored the importance of considering cultural and institutional factors when implementing IPE initiatives. Score variability across countries and academic years reflected differences in curriculum exposure, institutional emphasis on teamwork, or cultural influences on how collaboration is perceived. Institutional differences in curriculum design, teaching methodologies, and exposure to interdisciplinary environments may help explain the variation in students’ IPE readiness across the five countries studied. The distribution of total RIPLS scores varied by country, with a notable spread in responses observed particularly in the Teamwork and Collaboration domain. Figure 2 presents a boxplot comparing RIPLS score distributions across five countries (see Table 5).

**Table 2:** Interprofessional skill items analysis.

Items	Mean	SD
I will be able to contribute to a healthcare team more effectively if I learn alongside other students.	4.04	1.14
Understanding clinical problems better will come from sharing my knowledge with other healthcare students.	4.28	0.99
Relationships would be improved by learning with healthcare students prior to certification.	4.19	0.93
Acquiring knowledge from fellow healthcare students prior to graduation could enhance connections following graduation.	4.21	0.83
It is recommended that students acquire communication and techniques from one another.	4.15	0.91
My perspective on other experts will improve as a result of shared learning.	4.17	0.82
Students must trust and respect one another in order for small-group learning to be successful.	4.12	0.82
Teamwork is a crucial ability that all healthcare students must acquire.	4.12	0.80
Learning from others will enable me to recognize my own limitations.	4.26	0.88
I want to avoid wasting time learning alongside other health profession students.	2.18	1.07
Students majoring in healthcare do not always need to take classes together.	2.38	1.04
Students in my department are the only ones who can learn clinical problem-solving techniques.	2.45	0.96
Shared learning with other healthcare students will help me to communicate better with patients and other professionals	4.02	0.89
If given the chance, I would be happy to collaborate on small-scale initiatives with other medical students.	3.88	0.95
Clarity regarding the nature of patient problems would be aided by shared learning.	3.85	0.94
Prior to certification, shared learning will improve my ability to work with others.	4.00	0.85
The primary role of therapists and nurses is to help physicians.	3.98	0.83
I am not sure what my professional role will be	2.93	0.96
I have to acquire much more knowledge and skills than other healthcare students	3.56	0.95

**Table 3:** Comparison of overall interprofessional skill scores based on colleges.

Interprofessional skills	Dentistry (*N* = 135)	Medical (*N* = 112)	Nutrition (*N* = 16)	Applied health science (*N* = 64)	Physiotherapy (*N* = 8)	*P*-value
Teamwork and collaboration	3.8 ± 1.3	3.9 ± 1.06	4.0 ± 0	4.8 ± 0.4	5.0 ± 0.0	<0.001
Negative professional identity	2.1 ± 1.05	2.5 ± 1.05	2.0 ± 0	1.8 ± 0.9	2.0 ± 1.8	<0.001
Positive professional identity	3.9 ± 0.9	3.8 ± 0.9	4.1 ± 0.34	4.5 ± 0.50	4.5 ± 0.9	<0.001
Roles and responsibilities	4.0 ± 0.8	3.7 ± 0.8	3.8 ± 0.68	4.4 ± 0.60	4.8 ± 0.4	<0.001

**Table 4:** Comparison of overall interprofessional skill scores based on academic years.

Interprofessional skills	1st year	2nd year	3rd year	4th year	5th year	6th year	Interns	*P*-value
Teamwork and collaboration	3.6 ± 1.9	4.1 ± 0.9	5 ± 0	3 ± 2.06	3.5 ± 0.87	4.3 ± 0.4	4.5 ± 0.5	<0.001
Negative professional identity	3.1 ± 1.6	2.2 ± 0.04	1.8 ± 1.3	1.5 ± 0.5	2.2 ± 0.8	1.9 ± 0.2	1.5 ± 0.5	<0.001
Positive professional identity	3.7 ± 0.50	4.1 ± 0.8	4.3 ± 0.7	3.3 ± 1.1	3.4 ± 0.8	4.1 ± 0.8	4.5 ± 0.5	<0.001
Roles and responsibilities	4 ± 0.2	3.9 ± 0.8	4.8 ± 0.3	4.1 ± 0.3	3.5 ± 0.8	4.08 ± 0.7	4.5 ± 0.5	<0.001

**Table 5:** Comparison of overall Interprofessional education perception and preparedness among health professional students based in different countries.

Interprofessional skills	Malaysia (*N* = 176)	Saudi Arabia (*N* = 56)	Pakistan (*N* = 56)	India (*N* = 17)	Nepal (*N* = 30)	*P*-value
Teamwork and collaboration	4.2 ± 0.7	3.4 ± 1.3	4.1 ± 1.3	4.9 ± 0.2	3.2 ± 1.5	<0.001
Negative professional identity	2.3 ± 1.1	2.3 ± 1.2	1.6 ± 0.48	2.8 ± 1.1	1.4 ± 0.5	<0.001
Positive professional identity	4.1 ± 0.6	3.6 ± 0.7	4.1 ± 0.9	4.1 ± 0.3	3.5 ± 1.6	<0.001
Roles and responsibilities	3.9 ± 0.6	3.7 ± 0.7	4.2 ± 0.6	4.5 ± 0.5	3.5 ± 1.6	<0.001

## 4 Discussion

Interprofessional education (IPE) has emerged as a pivotal method in healthcare education, facilitating collaboration among students from diverse health professions. IPE plays a critical role in preparing health professional students to work collaboratively on diverse healthcare teams. Understanding students’ expectations, barriers, preparedness, and perceptions regarding IPE is essential to enhance educational strategies and improve healthcare delivery (16, 18, 19).

Regarding the expectations of IPE, health professional students generally expect it to improve their collaborative skills, enhance their understanding of different professional roles, and ultimately lead to better patient outcomes. Students anticipated that IPE would provide practical teamwork skills, which are vital in today’s complex healthcare environment (20). Research indicated that students who were engaged in IPE expected to gain insights into the perspectives and responsibilities of other health professionals, fostering mutual respect and understanding. However, despite these positive expectations, several barriers hindered the effective implementation of IPE. These included institutional challenges, such as siloed curricula that limit opportunities for interdisciplinary learning, logistical issues such as scheduling conflicts between programs (16, 18), and attitudinal barriers, with some students holding preconceived notions about other professions, which can lead to reluctance to collaborate (21). Addressing these barriers was crucial to fostering a collaborative educational environment.

Preparedness for IPE varied among students and was often influenced by their prior experiences and educational backgrounds. Students exposed to teamwork in clinical settings felt more prepared for IPE activities, while those lacking such experience expressed anxiety regarding collaborative tasks. Institutions can enhance preparedness by integrating IPE into training, allowing students to build confidence and skills progressively (22).

Perceptions of IPE were generally positive, with students recognizing its importance in their future practice. However, perceptions varied based on discipline and past experiences. For instance, nursing students viewed IPE as more beneficial than medical students, who felt that their training was more individualized (16, 18). Furthermore, positive experiences in IPE led to greater appreciation of collaborative practice, while negative experiences reinforced existing biases. Thus, fostering a supportive environment during IPE activities is essential for shaping favorable perceptions.

This study explored the readiness for IPE among students across several health science programs in selected countries, highlighting the various dimensions and implications of this educational approach. IPE aims to prepare students for collaborative practice by fostering essential competencies in communication, teamwork, and an understanding of professional roles and responsibilities. Our findings revealed high levels of readiness among medical and dental students from India, Pakistan, and Malaysia, affirming previous literature suggesting positive attitudes and improved knowledge exchange through IPE (19). It is important to note that the sample sizes from each country were not evenly distributed, with a disproportionately larger number of participants from Malaysia. This imbalance may have influenced the observed differences in readiness and perceptions across countries; therefore, cross-country comparisons should be made cautiously. Therefore, results from India (*N* = 17) and Nepal (*N* = 30) should be interpreted cautiously due to small sample sizes, which may limit generalizability.

The variation in students’ readiness and perceptions across countries may be partly attributed to cultural and institutional differences. For instance, in some contexts, hierarchical structures in healthcare education or a lack of curricular emphasis on teamwork may hinder openness toward interprofessional collaboration. In contrast, institutions that promote early clinical exposure and interprofessional engagement may foster more positive attitudes. These contextual factors should be considered when interpreting cross-country comparisons and in designing future IPE programs.

The integration of IPE into curricula is critical for enhancing student preparedness for collaborative healthcare delivery. Our study indicated a need for structured explanations of professional roles within healthcare institutions, which could help address uncertainties among students about their future responsibilities (23). Tailored educational interventions, such as reflective exercises and simulated experiences, could further optimize the learning outcomes of IPE across diverse student populations (22, 24).

Unlike some previous studies, our findings did not reveal significant effects of gender or age on readiness for IPE (21, 25, 26). This may be due to the balanced sex distribution and narrower age range within our participant cohort, suggesting potential demographic variations across studies (27). Our findings aligned with a study conducted in Oman, where nursing students, a predominantly female group, showed no significant differences based on demographic variables such as age, sex, or previous experience with IPE approaches (28). Raising awareness among health care students about the need for IPE, its benefits, and the rationale behind it attracted a larger number of students. Additionally, forming interdisciplinary teams and encouraging students to share their perspectives were feasible strategies for implementing IPE. Sessions on familiar topics, based on student and teacher availability, facilitated this. It is crucial for teachers to possess comprehensive knowledge of the topic, as suggested by students.

While this study provides valuable insights into students’ readiness and perceptions regarding interprofessional education (IPE), several limitations should be considered when interpreting the findings. In addition, informal feedback gathered during workshops revealed enthusiasm for collaborative IPE strategies such as role-play and small group projects, though some students expressed concerns about scheduling and unclear roles. There is a potential for selection bias, as the data were collected from a limited number of countries, and the results may not be generalizable to students at other institutions in different countries (29, 30). The nature of the study design may have affected the outcome due to selection bias (i.e., students interested in IPE could have been more motivated to answer) and the inability to measure interpersonal confounders, such as previous degrees and professional activities (29). Additionally, the self-reported questionnaires, such as the RIPLS, may have introduced social desirability bias, as students may have provided responses they felt were socially acceptable rather than entirely accurate (29, 30). Furthermore, the cross-sectional design of this study limits causal interpretations, and future research could benefit from longitudinal designs to assess the sustained impact of IPE on students’ professional development over time (29, 30). Finally, the lack of gender reporting by some students introduces the potential for gender bias in the results; therefore, future studies should aim for more balanced and accurate demographic data (29). Although interactive workshops were conducted to familiarize students with the IPE framework, no formal qualitative data, such as open-ended responses or reflective feedback, was collected. The lack of qualitative insights limits our ability to explore students’ deeper perceptions, attitudes, and personal experiences regarding interprofessional collaboration and learning.

Despite these limitations, the study provided several important recommendations for improving interprofessional education (IPE) across health professional programs. Therefore, we strongly recommend integrating IPE early in the curriculum to nurture interprofessional competencies from the outset of healthcare training (30). Moreover, IPE programs should be adapted to fit the cultural and institutional contexts of different countries, as our results show significant variations in students’ readiness based on their geographical location (31). Tailoring interventions such as group reflective exercises, simulation-based learning, and collaborative projects can further enhance students’ practical, real-world skills in interprofessional settings. Finally, future research should prioritize longitudinal studies to assess the long-term impact of IPE on professional development and collaboration in clinical practice.

## 5 Conclusion

This study provides actionable insights for health profession educators and policymakers. First, IPE should be introduced early in the curriculum to nurture collaborative competencies at the beginning of professional training. Second, the programs should be contextually adapted to local curricular and cultural settings to ensure relevance and acceptance. Third, longitudinal evaluation is needed to assess the sustained impact of IPE initiatives on clinical practice and interprofessional collaboration. This is one of the few recent multicenter studies from developing countries examining health professional students’ readiness for IPE, thereby contributing new evidence to a globally underrepresented context.

## Data Availability

The datasets presented in this study can be found in online repositories. The names of the repository/repositories and accession number(s) can be found in the article/Supplementary material.
